# Multiple Kernel Learning Captures a Systems-Level Functional Connectivity Biomarker Signature in Amyotrophic Lateral Sclerosis

**DOI:** 10.1371/journal.pone.0085190

**Published:** 2013-12-31

**Authors:** Tomer Fekete, Neta Zach, Lilianne R. Mujica-Parodi, Martin R. Turner

**Affiliations:** 1 Department of Biomedical Engineering, Stony Brook University, New York, New York, United States of America; 2 Biological Basis of Behavior Program, University of Pennsylvania, Philadelphia, Pennsylvania, United States of America; 3 Nuffield Department of Clinical Neurosciences, University of Oxford, Oxford, United Kingdom; Hangzhou Normal University, China

## Abstract

There is significant clinical and prognostic heterogeneity in the neurodegenerative disorder amyotrophic lateral sclerosis (ALS), despite a common immunohistological signature. Consistent extra-motor as well as motor cerebral, spinal anterior horn and distal neuromuscular junction pathology supports the notion of ALS a system failure. Establishing a disease biomarker is a priority but a simplistic, coordinate-based approach to brain dysfunction using MRI is not tenable. Resting-state functional MRI reflects the organization of brain networks at the systems-level, and so changes in of motor functional connectivity were explored to determine their potential as the substrate for a biomarker signature. Intra- as well as inter-motor functional networks in the 0.03–0.06 Hz frequency band were derived from 40 patients and 30 healthy controls of similar age, and used as features for pattern detection, employing multiple kernel learning. This approach enabled an accurate classification of a group of patients that included a range of clinical sub-types. An average of 13 regions-of-interest were needed to reach peak discrimination. Subsequent analysis revealed that the alterations in motor functional connectivity were widespread, including regions not obviously clinically affected such as the cerebellum and basal ganglia. Complex network analysis showed that functional networks in ALS differ markedly in their topology, reflecting the underlying altered functional connectivity pattern seen in patients: 1) reduced connectivity of both the cortical and sub-cortical motor areas with non motor areas 2)reduced subcortical-cortical motor connectivity and 3) increased connectivity observed within sub-cortical motor networks. This type of analysis has potential to non-invasively define a biomarker signature at the systems-level. As the understanding of neurodegenerative disorders moves towards studying pre-symptomatic changes, there is potential for this type of approach to generate biomarkers for the testing of neuroprotective strategies.

## Introduction

Amyotrophic lateral sclerosis (ALS) is a neurodegenerative disorder classically characterized by a loss of upper motor neurons of the motor cortex and corticospinal tract, and lower motor neurons of the brainstem nuclei and spinal cord anterior horns. A clinical, pathological and genetic overlap with frontotemporal dementia (FTD) is now recognized. The median survival is 3 years from symptom onset, but clinical and prognostic heterogeneity are well recognized. There is no highly effective disease-modifying therapy despite numerous trials [1]. Biomarkers would have potential for the more rapid and objective assessment of efficacy of therapeutic interventions in established disease, but also for identification of pre-symptomatic changes in those known to be at high risk of ALS [2]. They might also provide evidence for specific disease mechanisms [3], and thus novel targets for therapeutic intervention.

Cerebral pathology in ALS has been long-recognized as widespread [4], despite the obvious clinical predilection for motor pathways. The mild cognitive impairments detectable in at least one third of ALS patients have clear overlap with some forms of FTD [5], and there is a shared pathological link in the form of cytoplasmic inclusions of TDP-43 found throughout motor, pre-motor, frontal and temporal lobe regions [6]. ALS appears to be a systems-level, network-based disorders [7], and a simplistic, region-of-interest, co-ordinate-based approach will not capture widespread interconnected brain activity.

Resting-state functional MRI (R-fMRI) reveals temporally correlated low-frequency spontaneous fluctuations in blood oxygen level-dependent (BOLD) MRI signal, originating from several widespread functionally-distinct networks [8], [9]. While it cannot be validated in the same way as very specific task-based MRI (e.g. a visual task and occipital lobe function), very distinct and consistent regional networks at rest appear to closely reflect those seen in activity-based studies [10]. Alterations in R-fMRI data have been observed in ALS patients [11]–[18]. We wished to test the hypothesis that a systems-level signature capturing the core of ALS pathology, despite its inherent clinical and prognostic heterogeneity, might be identifiable using R-fMRI data.

## Methods

### Subjects

Patients were recruited from the Oxford Motor Neuron Disease Care and Research Centre as part of the Oxford Study for Biomarkers in Motor Neuron Disease (‘BioMOx’). All were apparently sporadic except one who reported a family history of ALS and FTD and was found to carry an expanded hexanucleotide repeat in *C9orf72*. All patients were diagnosed by experienced ALS neurologists according to standard criteria [19], [20], and all were either limb or bulbar in symptom onset. None were demented. Twenty-five patients overlapped with those used in a previously published study [15].

All participants underwent clinical examination on the day of study (M.R.T.), and were under active follow-up. Functional status was measured using the revised ALS Functional Rating Scale (ALSFRS-R, maximum score 48, falling with increasing disability) [21]. Disease duration was calculated from symptom onset to scan date in months. A rate of disease progression was calculated as (48 minus ALSFRS-R)/disease duration [22].

Healthy controls similar to the patients in age, gender, education and handedness for writing, were scanned under an identical MRI protocol. Ethical approval for all procedures was obtained prior to study from the South Oxfordshire Research Ethics Committee (08/H0605/85), and written informed consent was obtained from all participants. All data collection was carried out in the UK.

### Image Acquisition

Scans were performed at the Oxford Centre for Clinical Magnetic Resonance Research using a 3T Siemens Trio scanner (Siemens AG) with a 12-channel head coil, and in line with consensus guidelines put forward by the 2010 Neuroimaging Symposium in ALS (NISALS) [23]. Whole-brain functional imaging at rest was performed using a gradient echo planar imaging sequence (repetition time/echo time = 3000/28 ms, flip angle = 89°, 3 mm isotropic resolution, 9 min acquisition time). For maximum consistency, subjects were instructed to close their eyes throughout this latter sequence, but to remain awake.

### Image Analysis

Standard preprocessing procedures were performed in SPM8 [24], including image realignment correction for head movements, normalization to standard 3×3×3 mm Montreal Neurological Institute space, and spatial smoothing with an 8-mm full width at half maximum Gaussian kernel. Head motion estimates indicated that movement did not exceed one voxel in any of the subjects. Similarly, employing a two sample *t-test,* no significant difference was found in average head translation [25] between patients and controls. *xjview* (http://www.alivelearn.net/xjview) was used to visualize whole brain data.

### Motor Network Correlation Analysis

Time series were extracted for all grey matter voxels. To eliminate confounds in the data, the average time series in the CSF and white matter voxels was computed using the masks provided with SPM8, and concatenated with the SPM movement parameter time series. Next, the first order derivatives were computed for the eight time series, and all 16 time series were regressed out of the grey matter data [26], [27]. Finally data were linearly de-trended. The resulting time series were filtered to the 0.03–0.06 Hz bandwidth [28] using a Butterworth filter (order 4).

Using the WFU pickAtlas [29], the average time course for motor processing areas –M1, supplementary motor cortex, basal ganglia and cerebellum - was extracted following the AAL atlas [30]. Motor time series were used as seeds to compute correlation maps to all gray matter voxels (one map for each seed). This resulted in 24 maps (12L+12R), thus given our voxel size using the AAL masks resulted in 54,130 gray matter voxels for each map, that is a total of 1,299,120 features for each subject. Each map was reduced in dimension using principal component analysis (PCA) across subjects by anatomical region-of-interest (ROI) to comprise 10 scores for each of the 116 AAL regions, leaving a total of 27,840 features for each subject (116×24×10). We chose not to scale the number of scores to initial mask size, as there is no definite correlation between the size of brain areas and their functional significance (e.g. cerebellum vs. amygdala). Finally, the reduced maps were concatenated and used as features for classification.

### Classification

Classification was carried out in NeuroClass (http://www.lcneuro.org/), utilizing block diagonal optimization [31], a multiple kernel learning (MKL; [32]) support vector machine (SVM) approach with recursive kernel elimination [33]. The exact classification procedure is described in greater detail in appendix S1. In MKL, features are divided to subgroups – in our case according to anatomical ROIs – and a kernel computed for each ROI. The resulting kernels are then summed using weights found via block diagonal optimization. The single resulting kernel is then used for SVM classification. RCK enables to recursively rank kernels (ROIs) according to their contribution to the norm of the weights learned by the SVM.

To assess the performance of the classifier as well as optimize for the SVM soft margin parameter and carry out ROI selection, a two-tiered ‘leave one out’ (LOO) cross validation was carried out. On each validation fold an additional LOO was carried out to select the optimal hyperparameters of the SVM, as well as determine the number of ROIs for which classifier accuracy on the validation data peaked. The indices of entire peak region were stored and used to evaluate performance on test examples. Accordingly when performance was evaluated on test data the results were averaged and a majority vote applied.

In each training fold, data were initially scaled using a *z* transform. The training data means and variances were used to similarly scale test data. Next, training data features were filtered using ANOVA to retain only 10% of features in each ROI. Finally, the resulting feature vectors were normalized to unit length, a standard preprocessing step in MKL [34]. A radial basis function (RBF) SVM was used and optimized for the soft margin parameter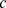 and RBF width 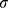 such that 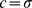 and 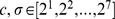. The classifying scheme above was first tested on an independent data set of *N* = 33 schizophrenia patients and controls that we had previously collected [35], and the parameters employed here were chosen according to the highest attained accuracy.

To verify the significance of the classification result a bootstrapping method was applied: a 1000 sets of permuted labels (with replacement) were generated such that the number of positive and negative examples in each of the original classes was balanced. Next, due to computational constraints, that is the run time necessary for complete optimization of classifier parameters and feature number, the classifier was applied with two simplifications; only the soft margin parameter selected by using the original labels was employed, and rather than optimizing for feature number, the maximal accuracy across features families was computed. Note that this in fact yields a positively biased null-distribution.

### Complex Network Analysis

To further probe the nature of the deviant motor functional connectivity in ALS we carried out complex network analysis [CNA, 36]. This subset of graph theory enables to explore the topology of the inter area functional connectivity matrix, and identify general properties of the functional connectivity pertaining to the network segregation, integration, resilience and centrality. To produce functional connectivity graphs, the preprocessed time series in the 0.03–0.06 Hz band were averaged by ROI. Next the cross correlation between the 116 signals was computed. The resulting functional connectivity graphs were then pruned from negative and auto correlations [36]. The resulting graphs were thresholded to retain a fraction α of the strongest connections. Initially the following threshold levels −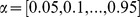 - were used to compute the small world ratio across subjects. Next, to select a threshold for subsequent analysis, we sought after the value that would give rise to topological network representations in which the small world properties would be the most pronounced. To that end we chose to optimize for an approximate measure of the signal to noise ratio of the small wordlness index: the ratio between the mean and standard deviation of the index across our sample. Accordingly, the threshold level that maximized the ratio 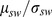, 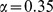 was chosen (Figure S1). The computation of both global and local CNA features was done using the toolbox reported in [36].

## Results

Participant characteristics (40 patients, 30 controls) are shown in Table 1. The groups did not differ significantly in age or gender balance.

**Table 1:** Participant demographics and clinical features.

	*Patients*	*Healthy controls*
*N*	40 (36 ALS, 4 PLS)	30
Mean age	55±9	50±14
Gender	24M:16F	13M:17F
Site of symptom onset	5B:15UL:20LL	NA
Disease duration (months)	51±55	NA
ALSFRS-R	34±5.8	NA
Rate of disease progression(ALSFRS points/month)	0.56±0.64	NA

ALSFRS-R: revised ALS Functional Rating Scale.

BO: bulbar.

LL: lower limb.

PLS: primary lateral sclerosis.

UL: upper limb.

### Classification

The classifier resulted in a *post hoc* accuracy of 87% (CV error 88%, sensitivity 88%, specificity 87%). The significance of the classification was verified through boot-strapping (see methods), suggesting that significance was at least *p*<0.001.

**Figure 1:**
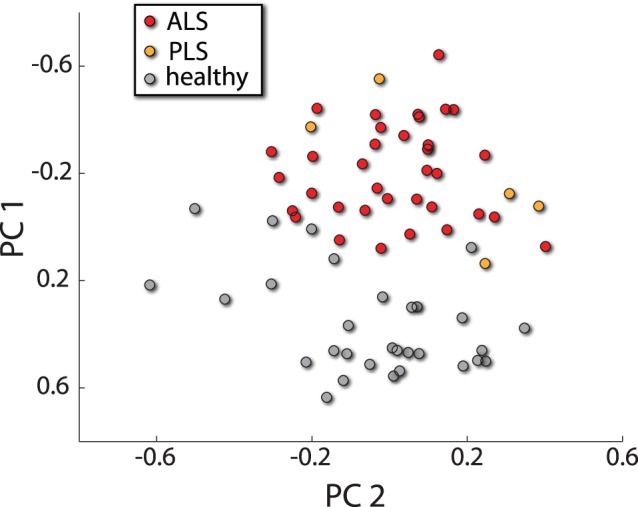
A graphical representation of classification results. The features and ROIs implicated by the classifier were embedded into 2D using principal component analysis.

The average number of ROIs to peak discrimination was 12.6±6.7 SD. The ROIs as ranked by the classifier across validation folds are shown in Table 2. To graphically represent the classification result, selected features from the top 13 ROIs implicated by the classifier were projected to 2D using principal component analysis (PCA – Figure 1). We compared the classification performance to classification based on the raw functional connectivity graphs, as well to local complex network measures based classification [31], in both cases using the same classifier build. However both alternative feature sets did not yield significant results.

**Table 2:** Anatomical regions contributing to peak classification.

*ROI*	*Participation*
Right supp. motor area	100%
Left hippocampus	100%
Right inferior temporal gyrus	97%
Left temporal Pole: superior gyrus	100%
Left middle frontal gyrus	100%
Left inferior frontal gyrus, orbital	99%
Left paracentral lobule	100%
Right paracentral lobule	100%
Right middle frontal gyrus, orbital	100%
Left anterior cingulate	86%
Vermis	97%
Left para-hippocampal gyrus	89%
Left cuneus	27%

Participation indicates the fraction of training folds in which a region was selected during optimization. Ranking reflects recursive kernel selection employed by the classifier. Rows highlighted in gray designate motor regions.

### Motor Network Connectivity

**Figure 2:**
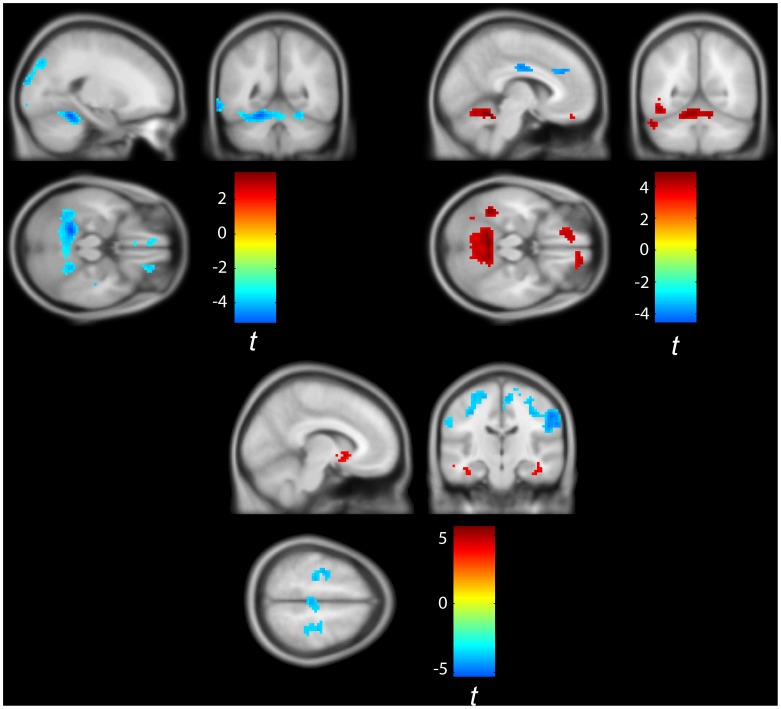
Aberrant functional connectivity in ALS. *Top left*: Group differences in gray matter functional connectivity to the right motor cortex (patients>control). Patients exhibited significant (*p*<0.01 random field corrected [61]) clusters of reduced connectivity in the 0.03–0.06 Hz frequency band mostly in the cerebellum, cuneus, rectus and fusiform gyri. *Top right*: Group differences in gray matter functional connectivity to the left Pallidum (patients>control). Patients exhibited significant (*p*<0.01 random field corrected) clusters of increased connectivity in the 0.03–0.06 Hz frequency band mainly in the cerebellum and rectus and reduced connectivity to cingulate and frontal areas as well as right SMA. *Bottom:* Group differences in gray matter functional connectivity to the left cerebellum (area 4/5 according to AAL classification - patients>control). Patients exhibited significant (*p*<0.01 random field corrected) clusters of decreased functional connectivity in the 0.03–0.06 Hz frequency band in the motor and somatosensory cortices together with clusters of increased connectivity mostly in the basal ganglia and cerebellum. Image was thresholded at *p* = 0.001 and cluster extent of 5 voxels.

As expected from the classification result, subsequent analysis of group differences in motor connectivity patterns (Welch 2-sample t-tests correcting for unequal variance, significance corrected using random field theory), indicated profound motor functional connectivity differences. Major group differences in the pattern of motor functional connectivity between the right primary motor cortex and left pallidum and cerebellum were observed (Figure 2, Figure S2). In general, patients exhibited hyper-connected sub-cortical motor networks spanning the basal ganglia and cerebellum, extending to involve additional cortical areas such as the infra-orbital gyrus rectus. This sub-cortical motor network exhibited reduced functional connectivity to motor cortices. Finally, the motor system at large exhibited reduced functional connectivity to frontal areas, occipital areas such as the cuneus and temporal lobe regions (fusiform and limbic cortices). In contrast we did not find a significant correlation between the clinical measures (ALSFRS-R, disease duration and progression) and the derived motor functional connectivity maps.

### Complex Network Analysis

**Figure 3:**
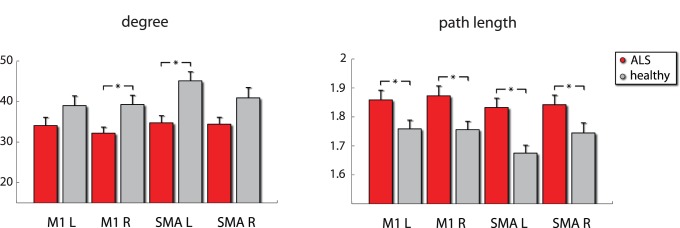
Altered topology of functional connectivity in the motor cortices in the 0.03–0.06 Hz frequency band. Right motor cortex and left supplementary motor cortex exhibited reduced degree i.e. extent of functional connectivity to other brain areas. Motor cortex and SMA exhibited increased path length bilaterally, indicating a reduced capacity for functional integration. *denotes *p*<0.05 corrected for ROI number (4) using an FDR approach.

**Figure 4:**
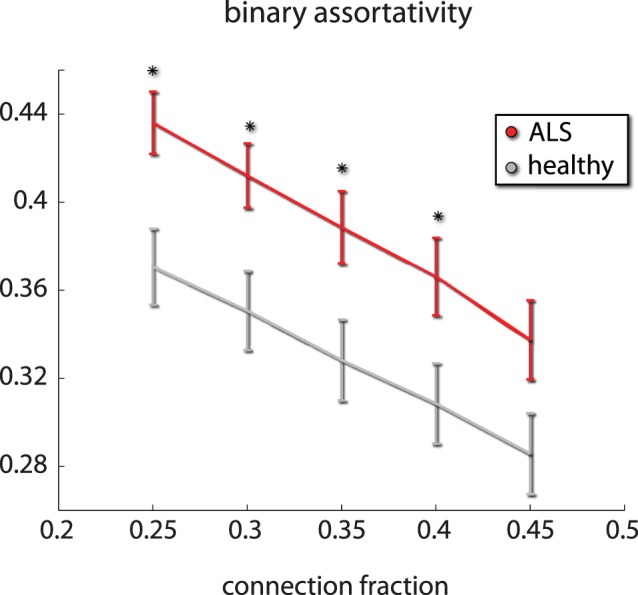
Complex network analysis. ALS results in global changes in the topology of inter-area functional connectivity. Inter area functional connectivity in the 0.03–0.06 Hz band exhibited increased assortativity i.e. correlation in degree between connected nodes in ALS patients. This reflects the existence of hyper-connected sub-cortical motor networks. We present the differences in the neighborhood of the chosen threshold (o.35). * denotes *p*<0.05 corrected using an FDR approach.

In the complex network analysis, local differences were found mainly in the motor cortices (Figure 3). The motor cortices of patients (SMA L M1 R) exhibited reduced node degree compared to controls, reflecting impoverished functional connectivity. Additionally, the motor cortices (SMA L/R M1 L/R) exhibited increased path length (average shortest path to all other network nodes) indicating compromised capacity for functional integration. Finally, global analysis revealed significant increase in patients in binary assortativity ([37]; figure 4) - a measure of the correlation in degree between connected nodes - reflecting the widespread increase in functional connectivity in both the basal ganglia and cerebellum, whereas we did not find differences in the small world properties of the functional networks between the two groups In contrast no correlation was found between the derived CNA measures and the clinical measures employed in this study (ALSFRS-R, disease duration and progression).

## Discussion

A multiple kernel learning classifier - block diagonal optimization [31] – was able to define a pattern of motor and extra-motor functional connectivity in the 0.03–0.06 Hz BOLD frequency range at rest. The signature derived enabled the accurate classification of a large group of ALS patients across a range of clinical sub-types, and healthy controls. Complex network analysis provided further evidence for a profound disintegration of cerebral network organization in ALS, including evidence of hyper-connected sub-cortical motor networks, with compromised functional coupling between motor and somatosensory cortices, the cerebellum and basal ganglia as well as widespread non-motor cortical networks.

### Previous Observations in the Resting-state in ALS

Studies of R-fMRI in ALS have also noted changes extending well beyond the primary motor cortex. The analysis methods have varied significantly, and the findings typically dichotomous, with regions of apparently increased as well as decreased functional connectivity (summarized in Table 3). Notwithstanding the need to optimize, standardize and ultimately harmonize R-fMRI analysis if it is to become a widely applicable tool, the common theme among the studies to date is that the ALS brain lesion represents a widespread system failure. An obvious hypothesis is that extra-motor changes, particularly increased functional connectivity, reflect compensatory processes in relation to the core structural disintegration within the motor cortex. However, at least two studies [13], [15] have specifically observed increased functional connectivity in those with faster rates of progression, at least raising the possibility that it has a more primary role in pathogenesis. Loss of interneuronal, inhibitory, cortical circuits is one hypothesis in this regard [38].

**Table 3:** Summary of resting-state functional MRI studies in ALS.

Participants	Methodology	Main finding	Clinical correlation	Reference
20 ALS 20 HC 9 LMNdisease controls	Whole-brain ICA	*Decreased* activation in sensorimotor anddefault-mode networks compared to bothHC and LMN disease controls	–	[11]
20 ALS 20 HC	Parcellation of PMC in topaired hemispheric ROIs	*Decreased* interhemispheric PMC FC	*Decreased* FC strength withincreasing hand strengthdisparity	[12]
12 ALS 12 HC	Whole-brain graph analysis,combined with DTI and SBM	*Decreased* interhemispheric PMC FC,callosal FA and PMC thickness	*Increased* PMC FC linked tofaster progression rate	[13]
26 ALS 15 HC	SMC ‘seed’ for wider whole-brain FC, combined with DTI	*Increased* FC between left SMC and rightcingulate/parahippocampal gyri andcerebellum; more widespread patternin those without CST DTI changes	Increased disability linked to*decreased* SMC FC	[14]
25 ALS 15 HC	Whole-brain dual-regressionanalysis of connectivityto a DTI-defined ‘ALS-specific’cortical network	*Increased* FC in regions of decreasedstructural connectivity spanningmotor, pre-motor andfrontotemporal cortices	*Increased* FC linked tofaster progression rate	[15]
20 ALS 20 HC	Whole-brain ICAcombined with VBM	*Decreased* activation in sensorimotor(PMC) and right fronto-parietal networks,with associated grey mattervolume reductions	Loss of the normal negativemodulation of age on default-mode network (specifically PCC)activity	[16]
20 ALS 15 HC	Whole-brain ICAcombined with VBM andcorrelated to ‘RSNtemplates’	1. Default-mode network: *decreased* FCof right orbitofrontal gyrusand *increased* FC of leftprecuneus; 2. Right fronto-parietal network:*decreased* FC of left anteriorinsula/inferior frontal cortex and*increased* FC of rightangular gyrus; 3. Left fronto-parietalnetwork: *increased* FC of left inferiorparietal lobule and middlecingulum	1. Default-mode network:*decreased* right precuneus FCwith increasing disability and*increased* left precuneus/angulargyrus FC with worse cognitivefunction; 2. Right fronto-parietalnetwork: *increased* right angulargyrus and left PCC FC with worsecognitive function	[17]
20 ALS 20 HC	Whole-brain amplitude oflow-frequency fluctuationcombined with VBM	*Decreased* amplitude fluctuation in inferioroccipital lobe, fusiform gyri and right post-central gyrus; *Increased* amplitude fluctuationin left middle frontal gyrus	*Increased* amplitude fluctuationswith increased disease durationand slower rate of progressionin left middle frontal gyrus	[18]

CST: corticospinal tract.

DTI: diffusion tensor imaging.

FC: functional connectivity.

HC: health controls.

ICA: independent component analysis.

LMN: lower motor neuron.

PCC: posterior cingulated cortex.

PMC: primary motor cortex.

RSN: resting-state network template.

SBM: surface-based morpometry.

SMC: supplementary cortex.

VBM: voxel-based morphometry.

### Evidence for a Wider, Clinically Silent Motor Network Pathology in ALS?

The patterns of altered functional motor connectivity in this study demonstrate the interconnected nature of the motor system. The loss of upper and lower motor neurons changes not only the functional connectivity within the motor cortex in which these upper motor neurons are embedded, but also in other motor areas such as the basal ganglia and the cerebellum, whose involvement is not visible as part of typical ALS symptomatology. In a previous fMRI study of motor task performance in ALS that revealed an increase in activation of the contralateral basal ganglia, this was interpreted as a recruitment in order to compensate for the limited primary motor cortex activation [39]. Similarly increased cerebellar activation was also found in a previous R-fMRI study [17], and changes have been observed in the cerebellar gray matter [40] and white matter longitudinally [41]. ALS associated with repeat expansions in *C9orf72* has characteristic cerebellar p62-positive cellular inclusions [42], and structural imaging changes in the cerebellum and thalami are prominent at least in cases of FTD associated with this mutation [43]. The present results suggest that cerebellar and basal ganglia network involvement may be more generalized and common to apparently sporadic ALS cases as well.

Patient-deviant functional connectivity was characterized by hyper-connected networks spanning non-cortical motor areas (notably putamen and cerebellum), as well as extra-motor cortical areas. These hyper-connected networks showed compromised connectivity to the motor cortices. In turn, not only did the motor cortices exhibit an intrinsic hypo–connectivity, but the motor system at large showed compromised functional connectivity to widespread cortical networks, reflected in complex network measures applied to the motor cortices. We speculate that this impoverished connection topology might be associated with reduced efficiency in the motor cortices. A previous study postulated that the brain has a “rich club” organization, involving dense interconnections formed by the major connectivity hubs in the brain which extend into the basal ganglia though the putamen [44]. The authors suggest this connectivity core may serve as a functional relay coupling the modular structures comprising the CNS. Thus, it is possible that the decoupling of the motor cortices from the basal ganglia in particular has far-reaching implications. Similar complexity analysis has been applied to ALS using DTI, rather than R-fMRI data. Studies have identified an impaired sub-network of reduced white matter connectivity centered around the primary motor cortex and paracentral lobule, but also specifically involving pallidum, middle frontal gyri and and hub regions represented by the posterior cingulate and precuneus [45], and with progressive disintegration over time [46].

### A Tool for Pre-symptomatic Studies?

The ALS R-fMRI signature defined in this study showed a striking lack of relationship to clinical variables. This has been a common finding in many ALS neuroimaging studies, across a range of techniques [47]. Clinical heterogeneity, with a bias towards atypical slowly-progressive cases in clinic-based studies like ‘BioMOx’, is an obvious concern. It is inherently challenging to capture ALS patients soon after symptom onset. The mean delay in diagnosis is resistant at one year [48], and only the most aggressive of cases present rapidly to specialists. However it also seems highly likely that, as in Alzheimer’s [49] and Huntington’s diseases [50], pathological events in ALS occur long before the onset of symptoms, and we suspect there is a significant ‘floor effect’ in pathological studies of symptomatic cases as a result, compounded by the fact the functional rating scales seem likely to reflect very downstream consequences of network dysfunction.

In what is an essentially sporadic disorder, a potential window on the earliest pathological changes is provided by the study of pre-symptomatic carriers of dominant genes linked to familial ALS [2]. In the few neuroimaging studies carried out in these individuals, there is already evidence for GABA-ergic cortical loss [51], white matter disorganization [52] [51], and spinal cord metabolite alterations [53]. Longitudinal R-fMRI studies in these individuals, using the unbiased approach outlined here has major potential to unravel the sequence of cerebral network changes, and to serve as biomarkers for future neuroprotective strategies.

### Prospects for Clinical Translation

These classification results add to a growing body of work targeted at exploring the potential of MRI-derived biomarkers to serve in the clinical setting, not only in ALS [54], but in other diseases such as depression, autism and schizophrenia [55]–[57]. Machine learning in the paradigm of MRI is intrinsically challenging. Data on the one hand are extremely high dimensional, yet the restrictions inherent to collecting fMRI data currently result in small sample sizes. Applying state-of-the-art machine learning algorithms greatly increases performance, especially in conjunction with feature selection as in this study. However, this comes at the cost of even larger data sets to allow for robust out of sample generalization, moving further away from the ultimate goal of single-subject analysis. The presence of numerous confounds known to influence functional connectivity-based measures, such as sex, age, motion [58], and ‘intelligence’ [59], not only calls for explicit modeling of demographic factors, but places even more demands on the volume and richness of data. Multi-center, international initiatives for data sharing and standardization are needed to attempt to address such issues, and these have begun in ALS [23] [60].

## Conclusions

These results demonstrate the unique potential of R-fMRI data to capture degeneration in ALS at the systems-level. The data can be acquired non-invasively in less than 10 minutes and in theory as part of a routine clinical work-up. The diagnostic biomarker value of the ALS resting-state signature will depend upon its performance in distinguishing disease mimics rather than healthy controls, and its monitoring value in its sensitivity to longitudinal change compared to clinical assessment. Meanwhile, this type of network analysis of cerebral functional connectivity confirms that ALS pathology has impact on widespread non-cortical areas whose involvement is not apparent on clinical examination.

## Supporting Information

Figure S1 **Small world properties of subject data.** *Left.* The small world ratio as a function of connection threshold across subjects. As shown by numerous previous studies, the topology of the functional network during rest exhibits small world properties. *Right.* The mean to variance ratio across subjects as a function of connection threshold. The threshold value 0.35 that maximized this ratio was employed for the subsequent analysis.
(EPS)

Figure S2 **Aberrant functional motor connectivity in ALS.** *Top*. Right primary motor cortex *Middle*. Left pallidum *Bottom*. Bottom right cerebellum. Image was thresholded at *p* = 0.001 and cluster extent of 5 voxels.
(TIF)

Appendix S1 **Multi kernel Block Diagonal optimization.** The classification pipeline employed in this study is described in greater detail.
(DOCX)
