# Supplementary figures and images for: Multiple Kernel Learning Captures a Systems-Level Functional Connectivity Biomarker Signature in Amyotrophic Lateral Sclerosis
Source: PLoS One. 2013 Dec 31;8(12):e85190. doi: 10.1371/journal.pone.0085190 (PMC3877396; doi:10.1371/journal.pone.0085190)

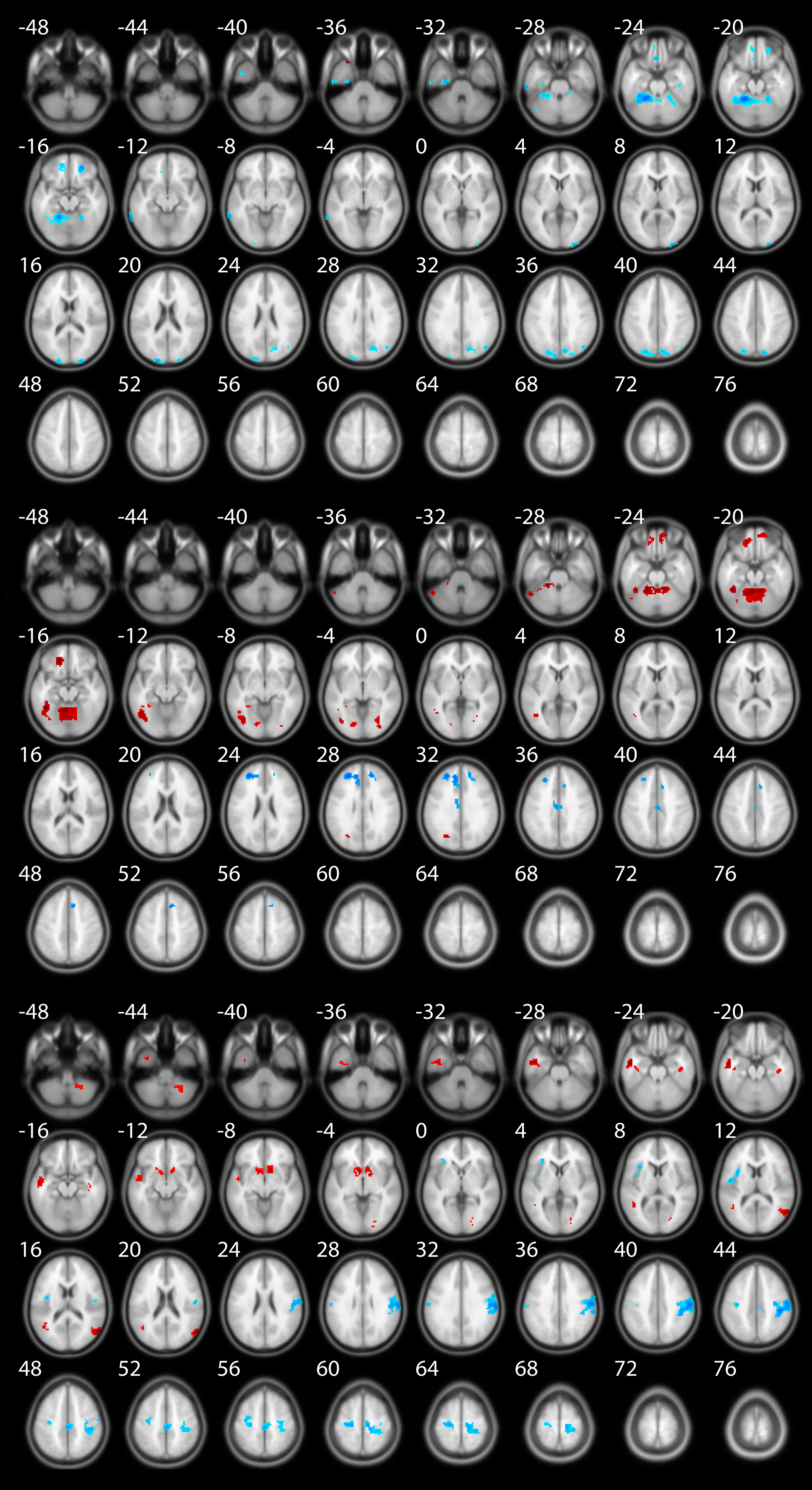

Supplement: Figure S2 — Aberrant functional motor connectivity in ALS. Top. Right primary motor cortex Middle. Left pallidum Bottom. Bottom right cerebellum. Image was thresholded at p = 0.001 and cluster extent of 5 voxels. (TIF) [file pone.0085190.s002.tif]
