# Supplementary material for: Multiple Kernel Learning Captures a Systems-Level Functional Connectivity Biomarker Signature in Amyotrophic Lateral Sclerosis
Source: PLoS One. 2013 Dec 31;8(12):e85190. doi: 10.1371/journal.pone.0085190 (PMC3877396; doi:10.1371/journal.pone.0085190)
Supplement: Appendix S1 — Multi kernel Block Diagonal optimization. The classification pipeline employed in this study is described in greater detail. (DOCX) [file pone.0085190.s003.docx]

APPENDIX S1: **Multi kernel Block Diagonal optimization**

The goal of a classifier is to label data in a test set (e.g., patient vs. control) according to information gleaned from learning data. All classification reported here was done using our NeuroClass (<http://www.lcneuro.org/>) - a publicly available Matlab toolbox for SVM-based classification. NeuroClass utilizes the LIB-SVM toolbox [[1](#_ENREF_1)] as its computational core.

**Feature scaling**. Prior to analysis, each feature was normalized across subjects in the training sample via a *z* transform and the estimated mean and standard deviation were used to scale the test data. Normalization is required to avoid driving results due to trivial scale differences between features, and the necessity to adapt classifier parameters to scale.

**Multi kernel Block Diagonal optimization (BDopt)**. Analysis of neuroimaging data inevitably results in a multitude of features (i.e., measures), which in turn could simply be fed into a classifier. However, there are substantial reasons for treating brain derived measures as *families* of features rather than individual features. First, grouping features according to anatomical ROI enables one to characterize a brain region in terms of its significance to the network as a whole, as well as to anatomically localize group differences in the case of pathology. Secondly, grouping features into families allows them to be "averaged", as explained in detail below, thus playing to the strengths of kernel based methods as far as feature number is concerned, as well as increasing the signal to noise ratio in scenarios in which the same information is latent across several of the families (e.g. several ROIs pertain to a condition of focus).

A general family of learning machines in which this can be naturally implemented is kernel sum machines, which is referred to as multiple kernel learning [[MKL, 2](#_ENREF_2)]. In MKL, each family of features is used to derive a kernel matrix: $(x_{i}^{r},x_{j}^{r})\mapsto k_{ij}^{r}$ where $x_{i}^{r}$is the feature vector originating from the *r^th^* anatomical region derived from the *i^th^* observation. Next, the kernels derived from all feature families are summed to produce a single kernel *K*, i.e.: $K=\sum_{r=1}^{R} w_{r}K^{r}$. This kernel can then be fed to a kernel based learning machine such as a support vector machine (SVM).

We developed a novel optimization method for deriving optimal weights for feature families: multi kernel block diagonal optimization (BDopt). The kernel matrix can be thought of as representing the degree of similarity between feature vectors. In BDopt, the optimal weight vector *w* is found by maximizing the ratio of within-class to between-class similarity. In the ideal scenario – which would lead to perfect classification – the similarity within class would be maximal, e.g. attain some maximal value *s* for each pair of observations belonging to the group, while the similarity between instances belonging to different groups would be virtually zero. If the data are organized according to class, that is, first the examples belonging to the first class, followed by the examples from the second class and so on, then the resulting kernel matrix would have the form of a block diagonal matrix: the matrix entries corresponding to within class similarity would attain the value *s,* while all other values would be zero (**Figure 1**). In general, this ideal similarity structure can be represented by a block-diagonal binary matrix. Thus *w_r_* can be found by minimizing the quadratic difference between the weighted sum of kernels and the block diagonal matrix *B*, i.e.: $\mathop{\mathrm{argmin}}\limits_{w} \left\|\sum_{r=1}^{R} w_{r}K^{r}-B\right\|^{2}$. This is, in fact, an ordinary least squares regression problem whose solution is given by $w_{opt}=\vec{k}^{T}(\vec{k}^{T}\vec{k})^{-1}\vec{b}$, where $\vec{k}^{T}$and $\vec{b}$ respectively denote the (column) vector representations of *K* and *B.* In the analysis described here, we applied BDopt to our data using an RBF kernel, i.e.: $K_{ij}^{r}=\exp\left(-\frac{\left\|x_{i}^{r}-x_{j}^{r}\right\|}{2\sigma^{2}}\right)$. The resulting SVM needs to be optimized for the soft margin parameter *C* and the RBF width $\sigma$. We applied the constraint$C=\sigma$ to curb prohibitive computation time.

**Feature selection.** In high dimensional classification, feature selection is crucial because increasing the dimensionality (number of features) leads to accumulation of not only signal, but also noise. Therefore, it is quite likely that for noisy features, the information they carry might be masked by noise as the number of features becomes excessive [[3](#_ENREF_3)]. This is true both for the number of feature families (ROIs), and for the dimensionality of each family. BDopt readily allows for feature selection at both levels.

To select features within each family, we used the ANOVA *F* statistic [[3](#_ENREF_3)], which allows the features to be ranked according to their discriminative power, given the training sample. After the features were ranked, only the top *k*% of the features in each family was retained.

To select feature families, recursive feature elimination (RFE, [[4](#_ENREF_4)]), can be applied to the weighted kernels (i.e., RCK; [[5](#_ENREF_5)]). In each iteration, an SVM is trained on the training sample, and the resulting weights are used to find the feature family that contributes the least to the classification. The quadratic norm of the SVM weights is given by $\left\|w_{SVM}\right\|^{2}=\alpha^{T}K\alpha=\alpha^{T}(\sum_{r} w_{r}K^{r})\alpha$. Given that the contribution of each weighted kernel to the SVM weights is$\alpha^{T}w_{r}K^{r}\alpha$, the least informative feature family is determined by $\mathop{\mathrm{argmin}}\limits_{r} \alpha^{T}w_{r}K^{r}\alpha$. This feature family can then be removed, and the process repeated. Note that this optimization results in a ranking of feature families according to their discriminative power.

**Cross-validation.** Due to the small number of subjects in typical human neuroimaging studies, the most suitable choice for cross-validation (CV) seems to be a leave one out (LOO) scheme. In LOO, each subject in turn is removed from the pool. Next, classification is carried out on the remaining subjects (training data). Finally, the model is tested on the withheld subject (test) data, after which the test data is returned to the pool. The result for all subjects is averaged to yield a success rate.

In classification of neuroimaging time series, the dimensionality (number of features) greatly exceeds the number of data points, which can lead to overfitting the data. Overfitting can be circumvented by taking several measures. First, it is critical to carry out feature selection in conjunction with cross validation, i.e., independently for each training sample (the data after removing example *i*), otherwise the classification results will be biased, resulting in an inflated success rate. Second, regardless of the choice of kernel, SVMs have at least one hyper-parameter that has to be optimized for. Therefore, an additional CV step is necessary to find both the optimal number of feature families (i.e., ROIs or graphs) and the optimal SVM parameters. This means that for each of the LOO training sets, an additional LOO CV is carried out to select the abovementioned parameters. After they are computed, the model can be retrained on the full LOO training set, and tested on the withheld data. Given the small number of samples in a typical neuroimaging classification scenario, it is common for several parameter combinations to yield the maximal success rate, making the choice of a specific optimal combination of parameters arbitrary. We must, therefore, retain multiple versions of the trained classifier, corresponding to the multiple optimal combinations of parameters, and let the test outcomes be decided by a majority vote among those.

1. Chang CC, Lin CJ (2011) LIBSVM: a library for support vector machines. ACM Transactions on Intelligent Systems and Technology (TIST) 2: 27.

2. Lanckriet GRG, Cristianini N, Bartlett P, Ghaoui LE, Jordan MI (2004) Learning the kernel matrix with semidefinite programming. The Journal of Machine Learning Research 5: 27-72.

3. Fan J, Fan Y (2008) High dimensional classification using features annealed independence rules. Annals of statistics 36: 2605.

4. Guyon I, Weston J, Barnhill S, Vapnik V (2002) Gene selection for cancer classification using support vector machines. Machine learning 46: 389-422.

5. Castro E, Martínez-Ramón M, Pearlson G, Sui J, Calhoun VD (2011) Characterization of groups using composite kernels and multi-source fMRI analysis data: Application to schizophrenia. Neuroimage.
